# Apple fruit periderms (russeting) induced by wounding or by moisture have the same histologies, chemistries and gene expressions

**DOI:** 10.1371/journal.pone.0274733

**Published:** 2022-09-29

**Authors:** Yun-Hao Chen, Jannis Straube, Bishnu P. Khanal, Viktoria Zeisler-Diehl, Kiran Suresh, Lukas Schreiber, Thomas Debener, Moritz Knoche

**Affiliations:** 1 Institute of Horticultural Production Systems, Fruit Science Section, Leibniz University Hannover, Hannover, Germany; 2 Institute of Plant Genetics, Molecular Plant Breeding Section, Leibniz University Hannover, Hannover, Germany; 3 Institute of Cellular and Molecular Botany (IZMB), Department of Ecophysiology, University of Bonn, Bonn, Germany; Universidade do Minho, PORTUGAL

## Abstract

Russeting is a cosmetic defect of some fruit skins. Russeting (botanically: induction of periderm formation) can result from various environmental factors including wounding and surface moisture. The objective was to compare periderms resulting from wounding with those from exposure to moisture in developing apple fruit. Wounding or moisture exposure both resulted in cuticular microcracking. Cross-sections revealed suberized hypodermal cell walls by 4 d, and the start of periderm formation by 8 d after wounding or moisture treatment. The expression of selected target genes was similar in wound and moisture induced periderms. Transcription factors involved in the regulation of suberin (*MYB93*) and lignin (*MYB42*) synthesis, genes involved in the synthesis (*CYP86B1*) and the transport (*ABCG20*) of suberin monomers and two uncharacterized transcription factors (*NAC038* and *NAC058*) were all upregulated in induced periderm samples. Genes involved in cutin (*GPAT6*, *SHN3*) and wax synthesis (*KCS10*, *WSD1*, *CER6*) and transport of cutin monomers and wax components (*ABCG11*) were all downregulated. Levels of typical suberin monomers (*ω*-hydroxy-C_20_, -C_22_ and -C_24_ acids) and total suberin were high in the periderms, but low in the cuticle. Periderms were induced only when wounding occurred during early fruit development (32 and 66 days after full bloom (DAFB)) but not later (93 DAFB). Wound and moisture induced periderms are very similar morphologically, histologically, compositionally and molecularly.

## Introduction

Russeting occurs on the skins of many fruit crop species, including of apples. In the smooth-skinned apple cultivars, russeting is perceived as a cosmetic impairment and so results in a quality downgrade in the packhouse, and so is the cause of significant economic loss for producers. In addition to cosmetic impairment, a russeted fruit skin is also more permeable to water vapor [1]. In this way, russeted fruit suffer increased rates of postharvest water loss in transit and storage and so a greater loss of packed weight and, hence, a yield loss at point of sale (apples are commonly priced on a per fresh weight basis). A further problem associated with increased postharvest water loss is an increased incidence of shrivel, so is a further cause of quality downgrade at point of sale.

In botanical terms, russeting represents the replacement of a relatively simple primary surface, an epidermis and hypodermis, by a more complex secondary surface, a periderm. This comprises a phellem, a phellogen and a phelloderm [2]. It is the suberized cell walls of the phellem that are responsible for the rough-textured, dull-brown appearance of a russeted fruit.

The etiology of russeting in fruit is complex and not entirely clear. Russeting can be triggered by mechanical damage caused by external biotic factors, such as feeding insects [3] or external abiotic factors such as abrasion–e.g., leaf rub [4] or the use of some agrochemicals [5]. More commonly, the causes of russeting are developmental, the first visible symptoms of the disorder being the appearance of cuticular microcracks [6–8]. Such microcracks result from various sources including from strain of the fruit surface caused by growth [9, 10] or exposure to surface moisture [11–15]. The latter includes exposure either to vapor-phase water (high humidity) or to liquid-phase water (fog, dew, rain) [16].

The formation of microcracks impairs the barrier properties of the cuticle. By a yet unknown mechanism, microcracks can then trigger the formation of a periderm in the hypodermis, just below the epidermis [17–20]. When fully formed, the periderm partially restores the barrier properties of the impaired primary surface [21]. From an evolutionary perspective, formation of a periderm is an effective repair mechanism [21].

A periderm is also formed in response to mechanical wounding of the fruit surface. Like microcracking, mechanical wounding impairs the barrier function of the cuticle. It is thus not unlikely, that the subsequent processes leading to periderm formation may therefore be the same. If this were the case, one would expect a periderm formed after wounding and after moisture induction of microcracking to have similar histologies, chemistries and gene expressions.

The objective of this study was to test the above hypothesis. We employed abrasion, using fine sandpaper, to induce periderm formation after wounding. This was compared to moisture induced periderms. Moisture often plays a role in the natural development of russeting. It can be induced experimentally by exposing the surfaces of a developing apple fruit to water [11, 12, 22].

## Material and methods

### Plant materials

‘Pinova’ apple (*Malus × domestica* Borkh.) grafted on M9 rootstocks were cultivated in an experimental orchard of the horticultural research station of Leibniz University Hannover at Ruthe (lat. 52°14’N, long. 9°49’E) according to current regulations for integrated fruit production. All fruit were selected to uniformity of size and color and freedom from defects, tagged and assigned to one of two treatments. A total of 125 trees in two adjacent rows were used for randomized sampling.

### Treatments and experiments

Fruit were subjected to one of two treatments. To **induce a wound periderm**, the fruit skin was gently rubbed in the equatorial plane with sandpaper (grit size 1000; Bauhaus, Mannheim, Germany). The opposite surface of the same fruit served as the control.

To **induce a moisture periderm** we followed the procedure established earlier [12]. Briefly, a tube cut from the tip of a disposable Eppendorf reaction tube (8 mm inner diameter, cut to ~ 17 mm in length) was mounted on the fruit surface using a non-phytotoxic, fast-curing silicone rubber (Dowsil^™^ SE 9186 Clear Sealant, Dow Toray, Tokyo, Japan). After curing, deionized water was injected into the tube through the hole in the tip. Thereafter, the hole was sealed with silicone rubber to prevent evaporative water loss. The tube was removed and resealed to the fruit surface every 2 d to avoid loosening as a result of surface expansion growth. Again, the opposite side of the fruit remained without treatment to serve as the control. Moisture exposure was terminated by carefully removing the tube and blotting the surface dry using a soft paper tissue. The attachment/detachment procedures themselves caused no visible damage to the fruit surface and, importantly, no russeting [12].

The following experiments were conducted:

A **time course study of periderm formation** following wounding or moisture treatments was conducted. Two batches of fruit were selected and tagged on the tree, 28 days after full bloom (DAFB). The first batch was wounded at 40 DAFB. The second batch was used for moisture induction, beginning at 28 DAFB. After 12 d of induction (at 40 DAFB), moisture treatment was terminated. For microcracking assessment, fruit were sampled at 0, 1, 2, 3, 4, 8 and 16 d after wounding or after termination of moisture treatment. For histology and analysis of gene expression, the sampling dates were 0, 2, 4, 8 and 16 after wounding or termination of moisture treatment.

The **compositions of periderms** induced by wounding, by moisture treatment, and that of a naturally russeted surface were investigated. In the subsequent season fruit reached a stage of development that was comparable to the time course study slightly earlier (at about 32 DAFB). Wounding was carried out at 32 DAFB and the fruit left on the tree until maturity (156 DAFB). The corresponding moisture treatment began at 31 DAFB and continued for 12 d. All fruit were harvested at maturity, photographed (Canon EOS 550D, lens: EF-S 18–55 mm, Canon Germany, Krefeld, Germany) and then either stored (sections of the fruit) in Karnovsky fixative or used for isolation of CMs and PMs, as described above.

The **developmental time course of periderm formation** following wounding was investigated by wounding fruit at 32 DAFB (‘early’), 66 DAFB (‘intermediate’) or 93 DAFB (‘late’). Samples for histology were taken 8 d after wounding and at maturity (156 DAFB).

### Methods

#### Microscopy

Fruit surfaces were inspected for microcracks following exposure to wounding and to moisture [12]. For this, a fruit was dipped in 0.1% (w/v) aqueous acridine orange (Carl Roth, Karlsruhe, Germany) for 10 min, then rinsed with deionized water and blotted dry using a soft paper tissue. The treated and the control areas were then inspected using fluorescence microscopy (MZ10F; GFP-plus filter, 440–480 nm excitation wave length, ≥510 nm emission wave length; Leica Microsystems, Wetzlar, Germany). Three to four digital images were taken (DP71; Olympus Europa, Hamburg, Germany) on six to ten fruit, at each sampling date.

Periderm development was assessed by microscopy using thin anticlinal sections prepared from tissue blocks embedded in paraffin [11]. Briefly, excised tissue blocks (about 6×3×3 mm, two blocks per fruit per tree) comprising the fruit skin and some of the outer flesh were excised from the treated and control areas and fixed in Karnovsky fixative [23]. Blocks were then rinsed in deionized water, incubated in 70% (v/v) aqueous ethanol overnight (16 h) and then dehydrated in an ascending series of ethanol (70, 80, 90 and 96% v/v, for 30 min each). The ethanol was then displaced by isopropanol (100%, 40 min ×2) followed by a xylene substitute (AppliClear; AppliChem, Münster, Germany; 40 min ×2). For paraffin infiltration, blocks were transferred to a 1:1 (v/v) mixture of paraffin/xylene substitute (Carl Roth; 40 min ×1) at 60 °C followed by fresh paraffin wax (40 min ×2). All the incubation steps were carried out at reduced pressure (10.8 kPa). Finally, the blocks were cast in paraffin wax in a metal mold. Embedded blocks were then cooled and stored at 4 °C pending analysis.

Thin sections (10 μm) were cut using a rotatory microtome (Hyrax M 55; Carl Zeiss, Oberkochen, Germany). Sections were transferred to glass microscope slides, dried at 38 °C for 16 h and then rehydrated in xylene substitute (10 min, ×2) followed by a descending series of ethanol (96, 80, 70 and 60%; v/v; 10 min each) and finally in deionized water (5 min, ×2). Sections were stained in the dark using Fluorol Yellow (0.005%, w/v; Santa Cruz Biotechnology, Texas, USA) dissolved in glycerol (90%, v/v; Carl Roth) and melted (~ 90 °C) polyethylene glycol 4000 (PEG 4000; w/v; Carl Roth) in a ratio of 1:1 for 1 h [24]. Following washing in deionized water, the sections were viewed under transmitted white light or incident fluorescent light (filter U-MWB; 450–480 nm excitation; ≥520 nm emission wavelength; Olympus) using a fluorescence microscope (BX-60 equipped with a DP 73 digital camera; Olympus). We examined a minimum of 50 sections per block. Two blocks from the same fruit represented a single replication and there were a minimum of three replications.

#### RNA extraction

Using a razor blade, thin patches of skin were excised from wounded, or moisture-treated, or un-treated (control) surfaces [22]. Skin patches from six fruit taken from six trees (one apple per tree) were collected within 15 min of picking and combined to obtain one replicate. The patches were immediately frozen in liquid nitrogen and held at -80 °C. For RNA extraction, the patches were ground in liquid nitrogen using a pestle and mortar. The RNA was extracted using the InviTrap Spin Plant RNA Mini Kit (STRATEC Molecular GmbH, Berlin, Germany) according to the manufacturer’s protocol. Genomic DNA was removed using the DNA-free^™^ Kit (Thermo Fisher Scientific, Waltham, Massachusetts, USA). The purity and quantity of the RNA was determined by measuring the absorbances at 230, 260 and 280 nm (Nanodrop 2000c; Thermo Fisher Scientific, Waltham, Massachusetts, USA). The RNA integrity was determined on a 1.5% agarose gel. Following dilution, the RNA samples (30 ng/μl) were converted into cDNA (LunaScript^®^ RT SuperMix Kit; New England Biolabs, Ipswich, Massachusetts, USA). A standard PCR with a pair of actin primers (EB127077) [25] and the DCSPol DNA polymerase kit (DNA Cloning Service, Hamburg, Germany) was carried out. The amplification was checked on a 1.5% agarose gel. Samples were stored at -80 °C pending further use.

#### Quantitative real-time PCR

Twelve key genes associated with periderm formation, and suberin, cutin and wax metabolism were analyzed by qPCR (for details see in S1 Table, Selected transcription factors and genes analyzed in the present study). These genes were selected because they all play key roles in moisture-induced periderm formation [22]. Specific primer pairs were designed on Primer3 (http://primer3.ut.ee/) (for details see in S2 Table, Primers sequences of the genes analyzed in the present study). A total of 900 ng of RNA in a 60 μl reaction vial were reverse transcribed into cDNA (LunaScript^®^ RT SuperMix Kit; New England Biolabs, Ipswich, Massachusetts, USA). Later, an 8 μl reaction volume containing 1 μl cDNA, primers (at 200 nM final concentration) and the Luna^®^ Universal qPCR Master Mix (New England Biolabs) were used to carry out quantitative real-time PCRs (QuantStudio^™^ 6 Flex Real-Time PCR System; Applied Biosystems, Waltham, Massachusetts, USA). Conditions were: one cycle at 95 °C for 60 s, 40 cycles at 95 °C for 15 s and 40 cycles at 60 °C for 60 s. A melting curve analysis (95 °C for 15 s, 60 °C for 60 s, 60 to 95 °C in 0.5 °C increments) was carried out after the final amplification.

All expression values were obtained from the QuantStudio^™^ Real-Time PCR Software v1.3 (Applied Biosystems) and normalized using the two reference genes *Protein disulfide isomerase* (*PDI*) (MDP0000233444) and *MdeF-1 alpha* (AJ223969.1) [26, 27].

#### Isolation of cuticular membranes and periderm membranes

Cuticular membranes (CMs) and periderm membranes (PMs) were isolated enzymatically [28] from skin patches of wounded or moisture treated fruit. Skins of naturally russeted or non-russeted fruit served as controls. Excised skin segments (ES) were punched using a biopsy punch (12 mm diameter; Acuderm, Terrace, FL, USA). The ES were incubated in an isolation medium containing pectinase (9%, v/v; Panzym Super E flüssig; Novozymes A/S, Krogshoejvej, Bagsvaerd, Denmark), cellulase (0.5% v/v; Cellubrix L.; Novozymes A/S) and NaN_3_ (30 mM) in 50 mM citric acid buffer adjusted to pH 4.0. The isolation medium was replaced periodically until CMs and PMs separated from the subtending tissues. The CMs and PMs were cleaned using a soft camel-hair brush, rinsed in deionized water, dried at 40 °C and kept above dry silica gel.

#### Quantification and identification of wax constituent by gas chromatography

Wax constituents of CM or PM were quantified and identified following the protocol of Baales et al. [29]. The CM and PM discs were cut into small fragments using a razor blade. Wax was extracted by incubating 0.5 to 1 mg of CMs and PMs in CHCl_3_ (5 ml per replicate) at room temperature on a horizontal rolling bench (RM; Ingenieurbüro CAT, M. Zipperer, Staufen, Germany) overnight. Tetracosane (100 μl of 10 mg tetracosane in 50 ml CHCl_3_) was added to the wax extract as an internal standard. The volume of the extract was reduced under a gentle stream of N_2_ at 60 °C. The extracted dewaxed CM and PM were removed from the extract and dried on Teflon discs for analysis of cutin and suberin monomers.

To avoid interference of wax constituents containing polar hydroxyl- and carboxyl groups with the GC column, waxes were derivatized by silylation. This process yields trimethylsilyl ethers and–esters of the respective constituents. Samples were derivatized at 70 °C for 45 min following addition of 20 μl BSTFA (N, O-bis(trimethylsilyl)-trifluoracetamid; Machery-Nagel, Düren, Germany) and 20 μl pyridine (Sigma Aldrich, Deisenhofen, Germany). Wax constituents were quantified using a gas chromatograph equipped with a flame ionization detector (GC-FID; CG-Hewlett Packard 5890 series H, Hewlett-Packard, Palo Alto, CA, USA; 307 column-type: 30 m DB-1 inner Diam. 0.32 mm, film thickness 0.2 μm; J&W Scientific, Folsom, CA, USA). For quantification, the peak areas were normalized using the tetracosane internal standard and the areas of the PMs or CMs.

For identification, a GC coupled to a mass spectrometer was used (GC-MS; Quadrupole mass selective detector HP 5971; Hewlett-Packard, Palo Alto, CA, USA). Individual constituents were identified by comparing the fragmentation patterns with published data and with our own data library. The number of replicates was two to three.

#### Quantification and identification of suberin and cutin monomers by gas chromatography

Suberin and cutin monomers were quantified and identified following the protocol of Baales et al. [29]. The extracted CMs and PMs were transesterified by incubation in 1 ml BF_3_/MeOH for 16 h at 70 °C. Thereafter, 20 μg of dotriacontane (100 μl of 10 mg dotriacontane in 50 ml CHCl_3_) was added as an internal standard. Depolymerization was stopped and 2 ml of saturated NaHCO_3_ was added.

The cutin and suberin monomers were extracted using CHCl_3_ (×3, 2 ml each). The CHCl_3_ phase was separated, washed with 1 ml HPLC grade water, dried with Na_2_SO_4_ and concentrated under a gentle stream of N_2_ at 60 °C. Samples were derivatized as described above. The monomers and constituents were quantified by GC-FID and identified by GC-MS as described above. The data were normalized relative to the internal standard and to the fruit surface area. The fragmentation patterns were compared with published data and our in-house library. The number of replicates was two to three.

### Data analysis

Total suberin, cutin and wax were calculated by summation of all individual constituents identified and quantified by gas chromatography. The PMs isolated from wounded, moisture treated or naturally russeted fruit often represent mixed polymers that comprise areas with patches covered by periderm adjacent to patches covered by cuticle and underlying epidermal and hypodermal cells. The area ratios may vary between replicates. Because suberin, cutin and wax share common monomers and constituents, it is impossible to attribute individual constituents obtained in the compositional analyses of these mixed polymers to either the cutin or the suberin fractions. However, in an earlier study we quantified the mass ratios for typical constituents of suberin from the trunk of ‘Pinova’ trees [22]. The constituents unique for suberin are the *ω*-hydroxy-C_20_, -C_22_ and -C_24_ acids. These *ω*-hydroxy-acids account for 17.6% of the total suberin. Using these constituents and the composition of a ‘pure’ native periderm, the composition of mixed PMs could be calculated and assigned to the PM. As pointed out by Straube et al. [22], the calculation is based on the assumption that the suberin composition of a ‘Pinova’ fruit PM is identical to that of the trunk periderm of the same cultivar. Due to the lack of PM-specific wax constituents, this calculation was not possible for the wax fraction.

Data are presented as means ± standard error (SE) of the means. Where error bars are not visible, they are smaller than data symbols. Data were subjected to analyses of analysis of variance, regression analysis or t-tests using the statistical software SAS^®^ Studio (SAS 9.4; SAS Institute, Cary, NC, USA). Significance of *P*-values at the 0.05 level is indicated by *.

## Results

**Fig 1:**
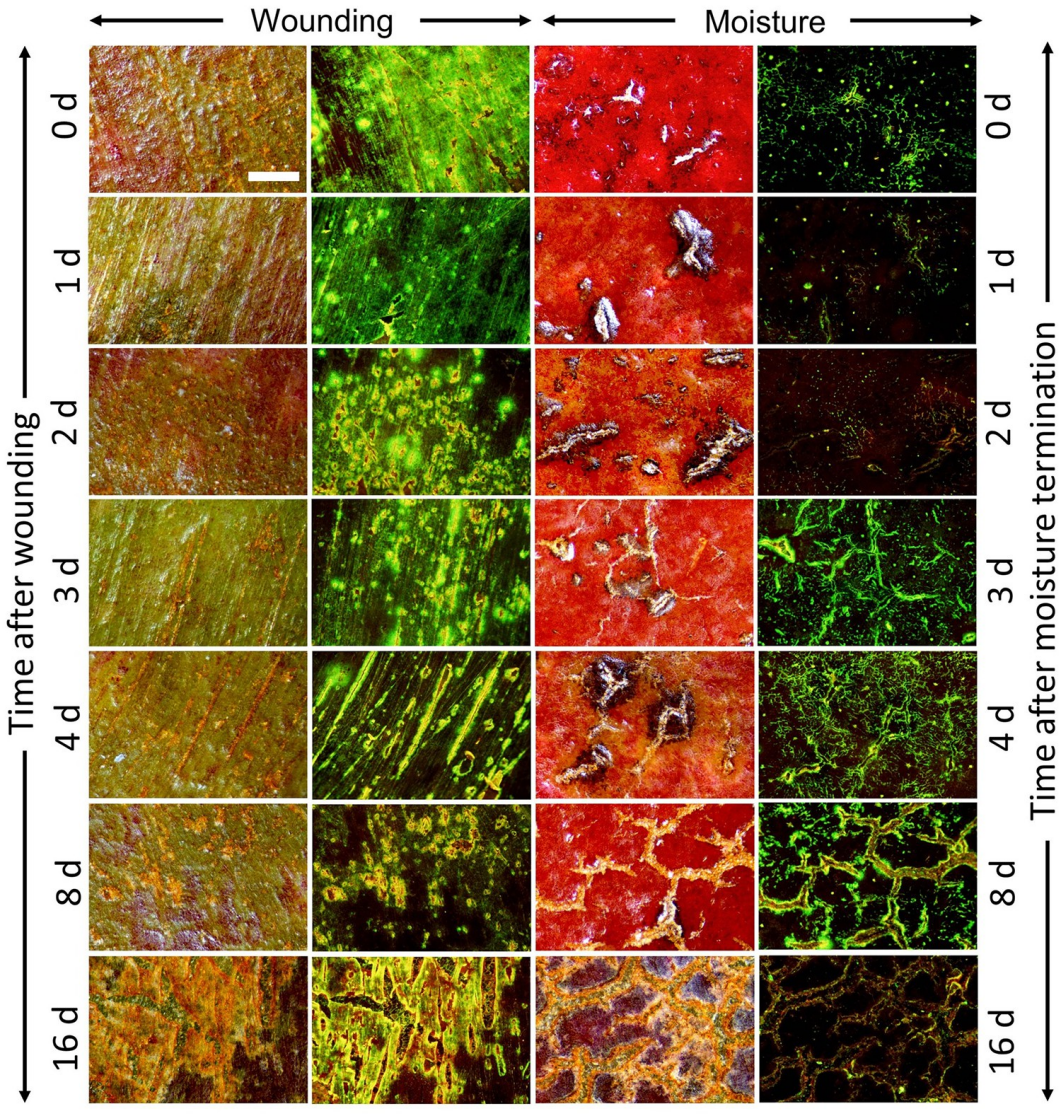
Time course of change in infiltration of ‘Pinova’ fruit skin patches following wounding by abrasion of the cuticle at 40 days after full bloom (DAFB) using fine sandpaper (‘Wounding’) or by exposure of the fruit skin to moisture for 12 d (‘Moisture’). Moisture exposure began at 28 DAFB. At 40 DAFB, moisture exposure was terminated and the time-course of change in infiltrated fruit surface area was established. Micrographs from the same surface area were taken under incident white light or incident fluorescent light. The green/yellow fluorescence resulted from localized penetration of the tracer acridine orange through microcracks in the cuticle into the underlying tissues. Scale bar equals 400 μm and is representative for all the images of the composite.

Wounding by abrading the skin of developing apple fruit resulted in numerous microcracks in the cuticle. The microcracks and the surrounding dermal tissue were infiltrated by aqueous acridine orange. As growth progressed after wounding, the microcracks widened (Fig 1). Microcracks also formed after a 12-d moisture treatment (Fig 1). Like the microcracks resulting from wounding, those caused by surface moisture treatment also traversed the cuticle as indexed by infiltration with aqueous acridine orange. In contrast to microcracks resulting from abrasion, those caused by moisture treatment were not straight and parallel to one another but followed the pattern of the anticlinal cell walls of groups of epidermal cells. Furthermore, moisture-induced microcracks branched at tricellular junctions.

**Fig 2:**
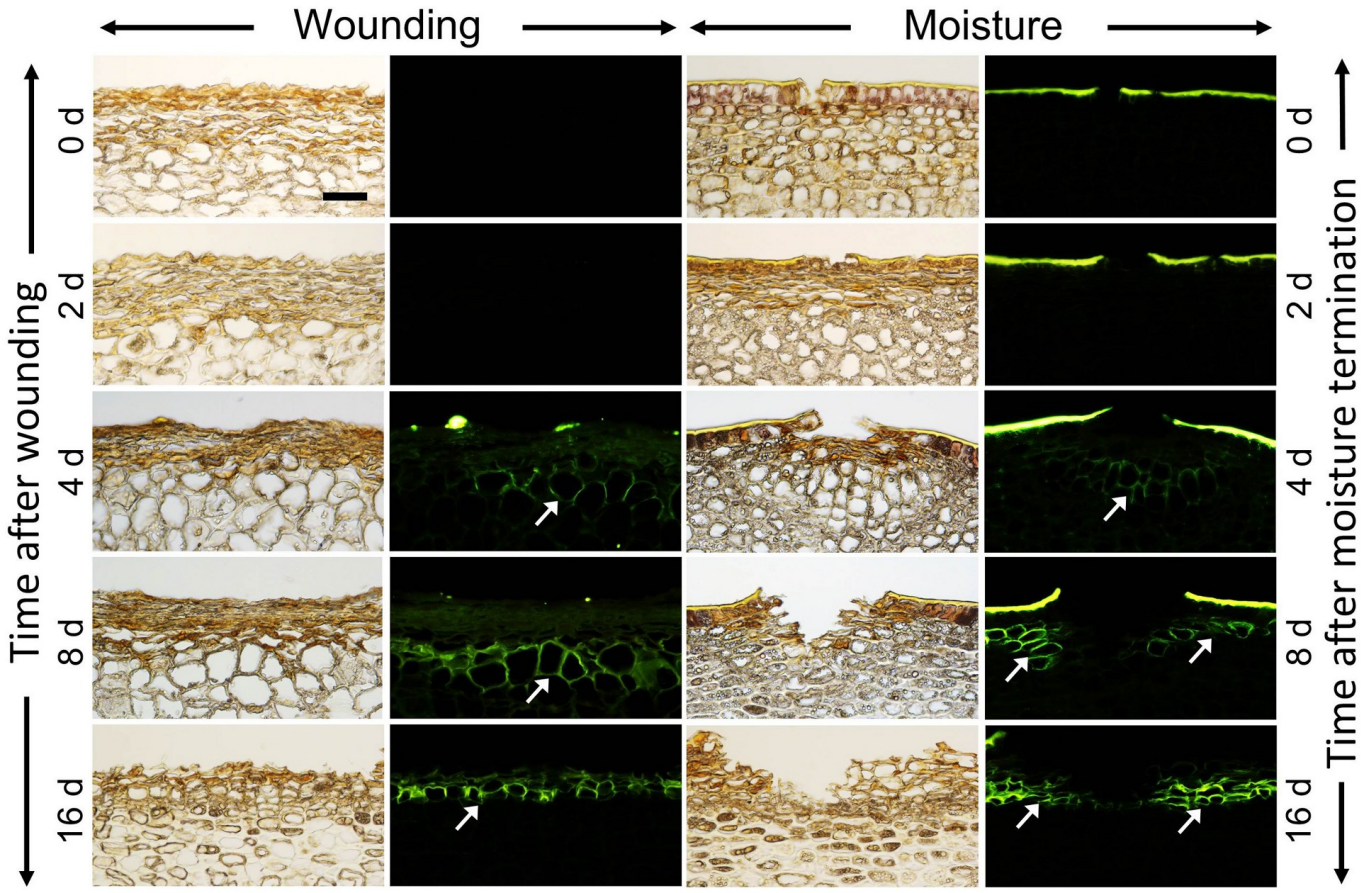
Time course of periderm development following wounding (left panel) and moisture exposure (right panel) of skin patches of ‘Pinova’ apple. Patches of skin were abraded 40 days after full bloom (DAFB) using fine sandpaper. Microcracks induced by surface moisture served as control (‘Moisture’). Here, the fruit surface was exposed to surface moisture for 12 d from 28 to 40 DAFB. Pairs of micrographs were taken under transmitted white light or incident fluorescent light (filter module U-MWB) after staining with Fluorol Yellow. Fluorol Yellow stains the cuticle and suberized cell walls. Scale bar equals 50 μm and is representative for all images of the composite.

Cross-sections of wounded apple fruit skins revealed browning and death of epidermal and some hypodermal cells shortly after abrasion (Fig 2). By 4 d after wounding, cell walls in the hypodermal cell layers began to suberize (marked with arrows) as indexed by staining with Fluorol Yellow. By 8 d after wounding, and even more so by 16 d, stacks of cells with suberized cell walls had formed that are characteristic of a periderm.

In cross-sections of moisture treated fruit skins of the same developmental stage, microcracks were present in the cuticle. These microcracks widened and the cuticle curled upwards as fruit growth continued, indicating the presence of considerable growth strain. At 4 d after termination of moisture treatment, the cell walls of the hypodermal cells below the microcrack began to suberize. By 8 d and 16 d after termination of moisture treatment, periderm formation had begun (Fig 2).

**Fig 3:**
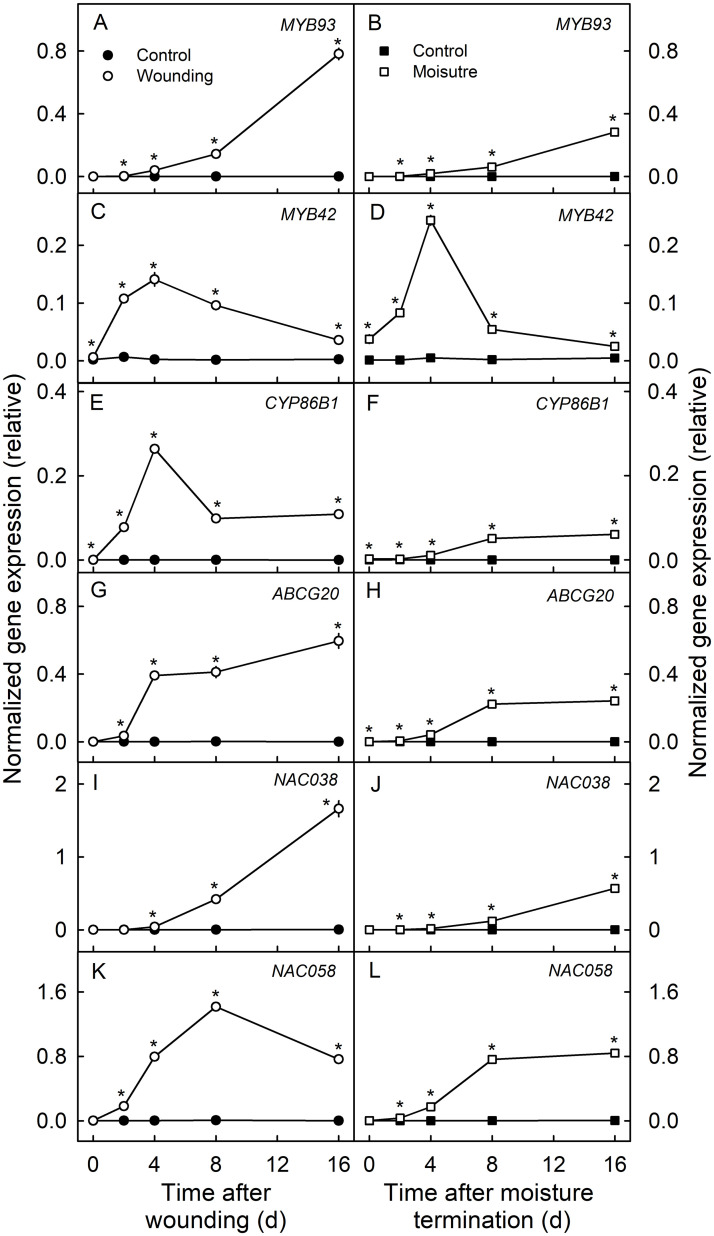
Time courses of change in the expressions of two transcription factors involved in the regulation of the synthesis of suberin (*MYB93*) and lignin (*MYB42*), a gene involved directly in the synthesis of suberin monomers (*CYP86B1*) and a gene involved in the transport of suberin monomers (*ABCG20*) and two uncharacterized transcription factors (*NAC038* and *NAC058*) in the skin of ‘Pinova’ apple fruit following wounding or following exposure of the fruit surface to moisture. Patches of fruit skin were wounded 40 days after full bloom (DAFB) by abrading the cuticle using fine sandpaper (‘Wounding’). For comparison, microcracks were induced by exposure of skin patches to surface moisture (‘Moisture’). Here, the fruit surface was exposed to surface moisture from 28 to 40 DAFB. Non-treated fruit served as the respective controls (‘Control’). Expression values are means ± SE of three biological replicates comprising six fruit each. The ‘*’ indicates significant differences between the wounded patch and its control or between the moisture exposed patch and its control, P ≤ 0.05 (Student’s t-test).

The two transcription factors involved in the regulation of the synthesis processes of suberin (*MYB93*) and lignin (*MYB42*), a gene involved directly in the synthesis of suberin monomers (*CYP86B1*) and a gene involved in the transport of suberin monomers (*ABCG20*) were all upregulated. The other two transcription factors (*NAC038* and *NAC058*), that do not yet have assigned functions, were also upregulated. Relative normalized expressions of *MYB42*, *CYP86B1* and *NAC058* were highest at 4 d or at 8 d but then decreased slightly at 16 d (Fig 3C, 3E and 3K) whereas the expressions of *MYB93*, *ABCG20* and *NAC038* increased continuously to 16 d (Fig 3A, 3G and 3I). The log fold changes in expression are provided in the S1 Dataset.

Very similar expression profiles, but at somewhat lower levels, were obtained in the moisture treated patches (Fig 3B, 3D, 3F, 3H, 3J and 3L). The only exception was the upregulation of *MYB42* in moisture treated fruit at 4 d after termination of moisture treatment. This exceeded that in the wounded fruit (Fig 3D).

**Fig 4:**
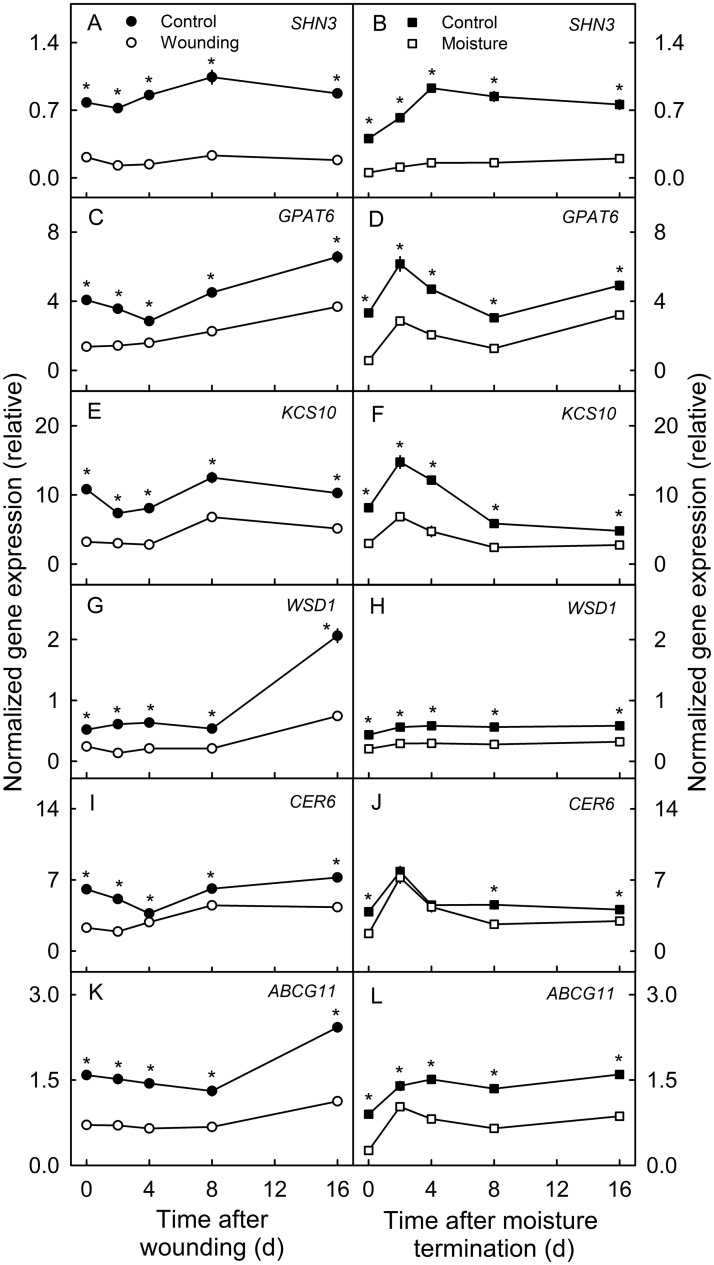
Time courses of change in the expression of genes involved in the synthesis of cutin monomers (*SHN3*, *GPAT6*) and wax constituents (*KCS10*, *WSD1*, *CER6*) and their transport (*ABCG11*) in the skin ‘Pinova’ apple fruit following wounding or following exposure of the fruit surface to moisture. Patches of fruit skin were wounded 40 days after full bloom (DAFB) by abrading the cuticle using fine sandpaper (‘Wounding’). For comparison, microcracks were induced by exposure of skin patches to surface moisture (‘Moisture’). Here, the fruit surface was exposed to surface moisture from 28 to 40 DAFB. Non-treated fruit served as control (‘Control’). Expression values are means ± SE of three biological replicates comprising six fruit each. The ‘*’ indicates significant differences between the wounded patch and its control or between the moisture exposed patch and its control, P ≤ 0.05 (Student’s t-test).

Genes involved in the synthesis of cutin (*SHN3*, *GPAT6*) and wax (*KCS10*, *WSD1*, *CER6*) and the transport of cutin monomers and wax components (*ABCG11*) were downregulated in both wounded and moisture treated skin patches (Fig 4). In general, the relative expressions were qualitatively and quantitatively similar in the wounded and moisture treated skin patches.

**Fig 5:**
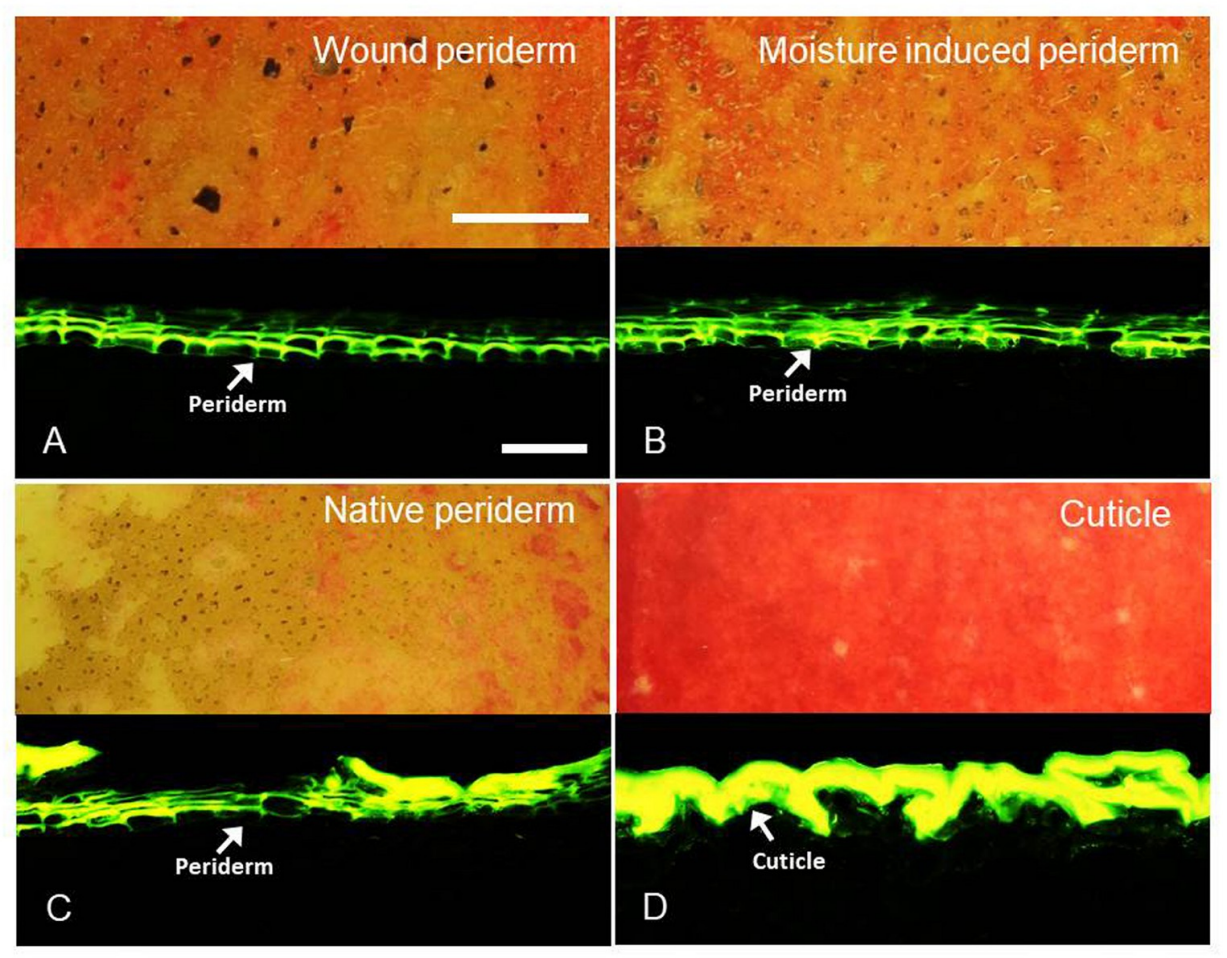
Cross-sections through patches of ‘Pinova’ apple fruit skin at the mature stage (156 days after full bloom (DAFB)) that had been wounded or exposed to surface moisture during early fruit development. Patches of fruit skin were wounded at 32 DAFB by abrading the cuticle using abrasive paper (‘Wound periderm’). For comparison, microcracks were induced by exposure of skin patches to surface moisture (‘Moisture-induced periderm’) from 31 to 43 DAFB. Non-treated naturally russeted surfaces (‘Native periderm’) and non-russeted surfaces served as control (‘Cuticle’). The cross-sections were stained with Fluorol Yellow. Scale bars in A 10 mm (upper) and 50 μm (lower). Bars are representative for all bright field and all fluorescence images of the composite.

Skin patches that were wounded or moisture treated for 12 d during early fruit development had formed a continuous periderm (marked with arrows) and developed a russeted surface by maturity. The periderms following wounding or moisture treatment were indistinguishable from the periderms of naturally russeted fruit of the same cultivar (Fig 5). At maturity, the skins of non-russeted patches had developed a thick cuticle (marked with arrows).

**Fig 6:**
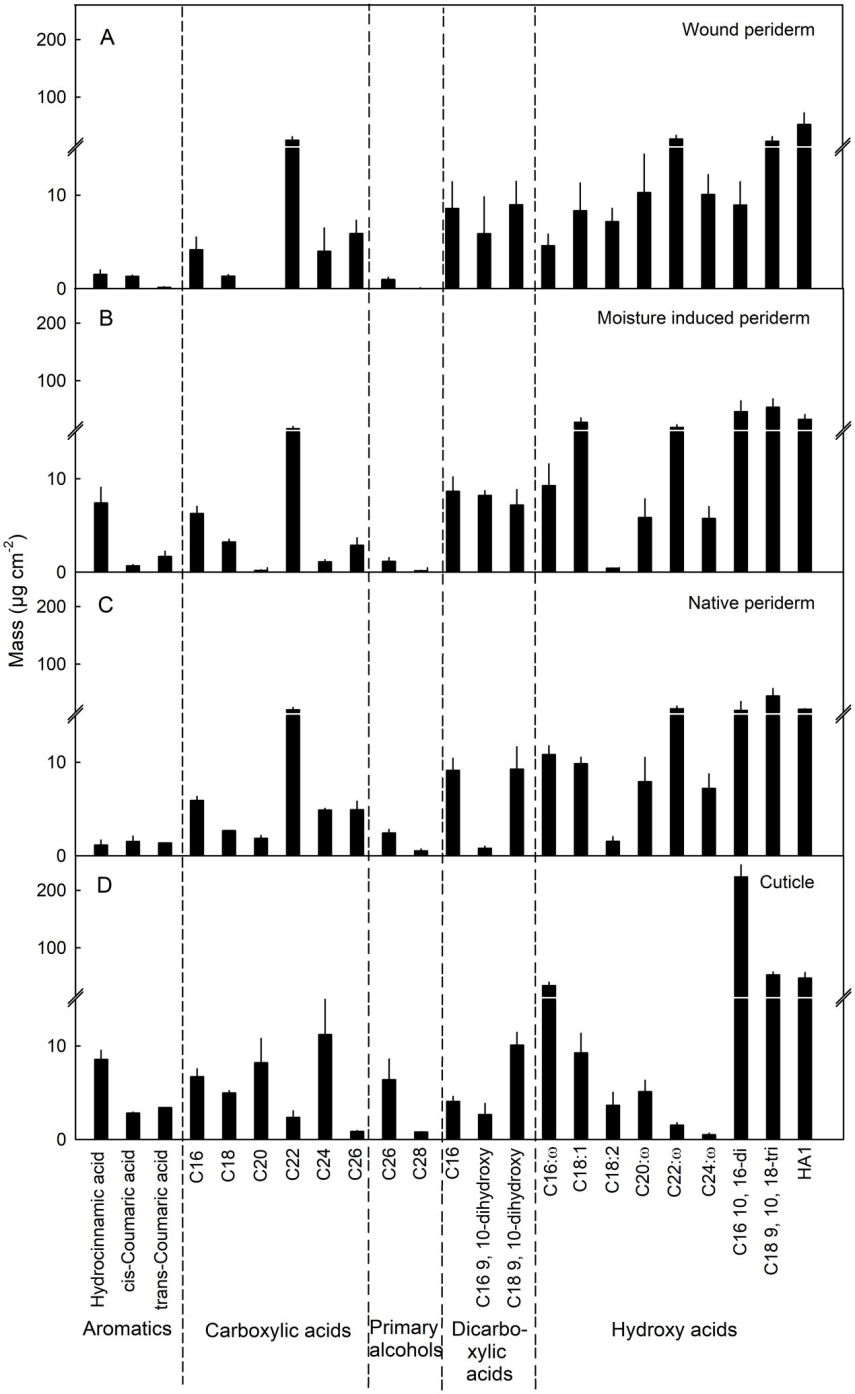
Composition of cutin and suberin of skins of mature apple fruit. Periderm formation in the fruit skin was induced during early development by abrading the cuticle using abrasive paper (‘Wound periderm’) (A) or by exposing the fruit skin to surface moisture for 12 d between 31 and 43 days after full bloom (DAFB; ‘Moisture-induced periderm’) (B). The treated patches of skin were excised at maturity 156 DAFB. Native periderm from naturally russeted fruit (C) and cuticles from non-treated non-russeted fruit served as controls (D). Data represent means ± SE of two to three replicates comprising periderms and cuticles of five fruit each. The data shown in (B) were taken from Straube et al. [22].

The monomer compositions of the periderms induced by wounding or by moisture treatment and that of the native periderm were very similar. The amounts of the typical suberin monomers *ω*-hydroxy-C_20_, -C_22_ and -C_24_ acids were very similar in all three periderms (wound induced, moisture induced, and native), and were significantly lower in the cuticle. The contents of carboxylic-C_22_ acid were also very similar in the three types of periderms but were much lower in the cuticle (Fig 6). Minor differences between the three types of periderms were: (1) The 9,10-dihydroxydicarboxylic-C_16_ acid was similar in wound and moisture induced periderms but significantly lower in native periderm. (2) The 1-hydroxy-C_18_ acid was present in higher amounts in moisture induced periderm than in wound and native periderm. (3) The 2-hydroxy-C_18_ acid content was higher in wound periderm than in moisture induced or in native periderm. (4) The hydrocinnamic acid was higher in moisture induced periderm than in the wound or native periderm. The abundances of this monomer were similar in the cuticle and moisture induced periderm (Fig 6).

**Fig 7:**
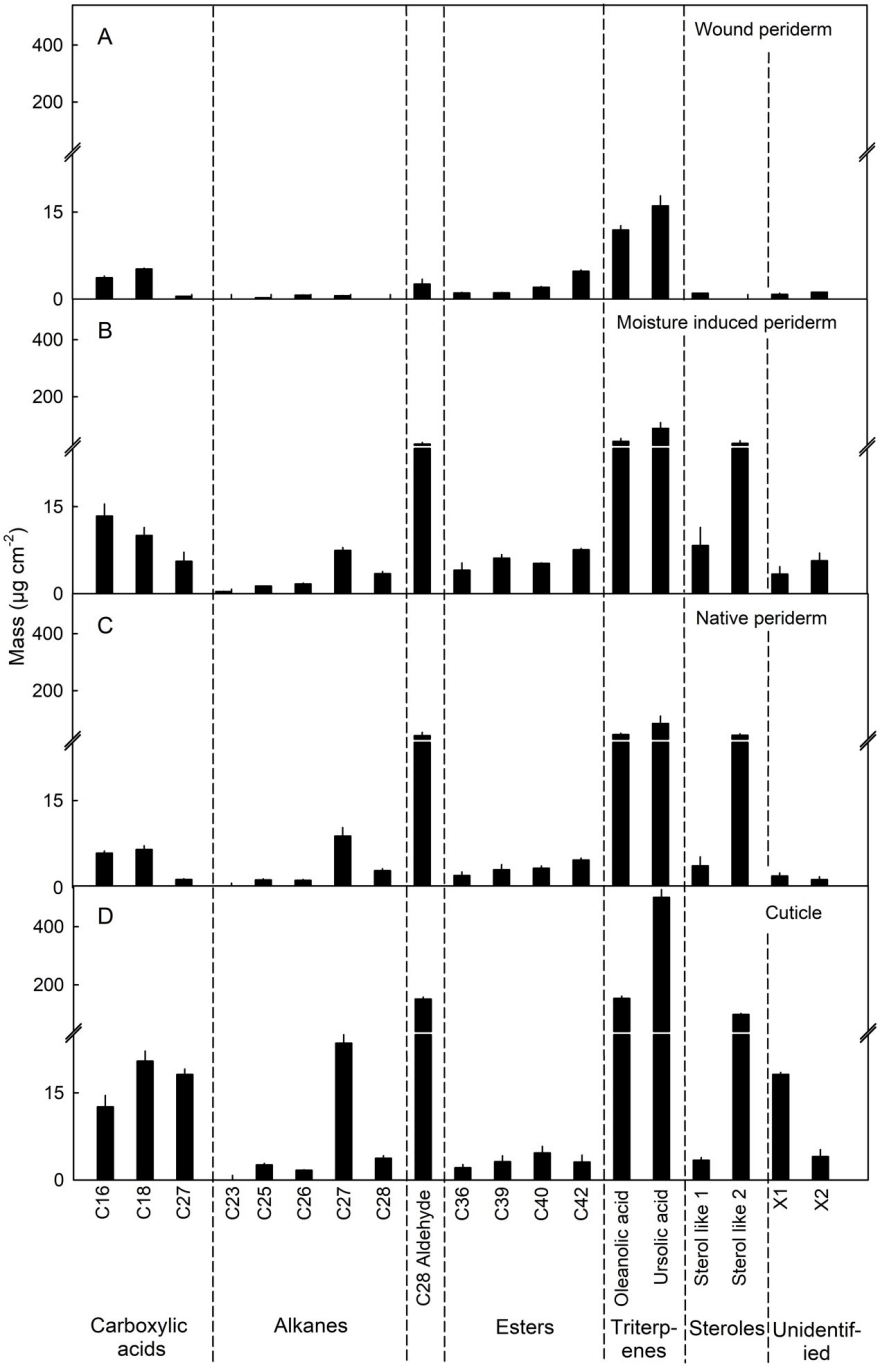
Wax constituents of the skins of mature apple fruit. Periderm formation in the fruit skin was induced during early development by abrading the cuticle using fine sandpaper (‘Wound periderm’) (A) or by exposing the fruit skin to surface moisture for 12 d between 31 and 43 days after full bloom (DAFB; ‘Moisture-induced periderm’) (B). The treated patches of skin were excised at maturity 156 DAFB. Native periderm from naturally russeted fruit (C) and cuticles from non-treated non-russeted fruit served as controls (D). Data represent means ± SE of two to three replicates comprising periderms and cuticles of five fruit each. The data shown in (B) were taken from Straube et al. [22].

Wax occurred in low amounts in the moisture induced and native periderm and was even lower in the wound periderm. The composition of wax was similar in the moisture induced and native periderm. Dominating wax components in moisture induced and native periderms and in the cuticle were C_28_ aldehydes, oleanolic and ursolic acids (Fig 7).

**Fig 8:**
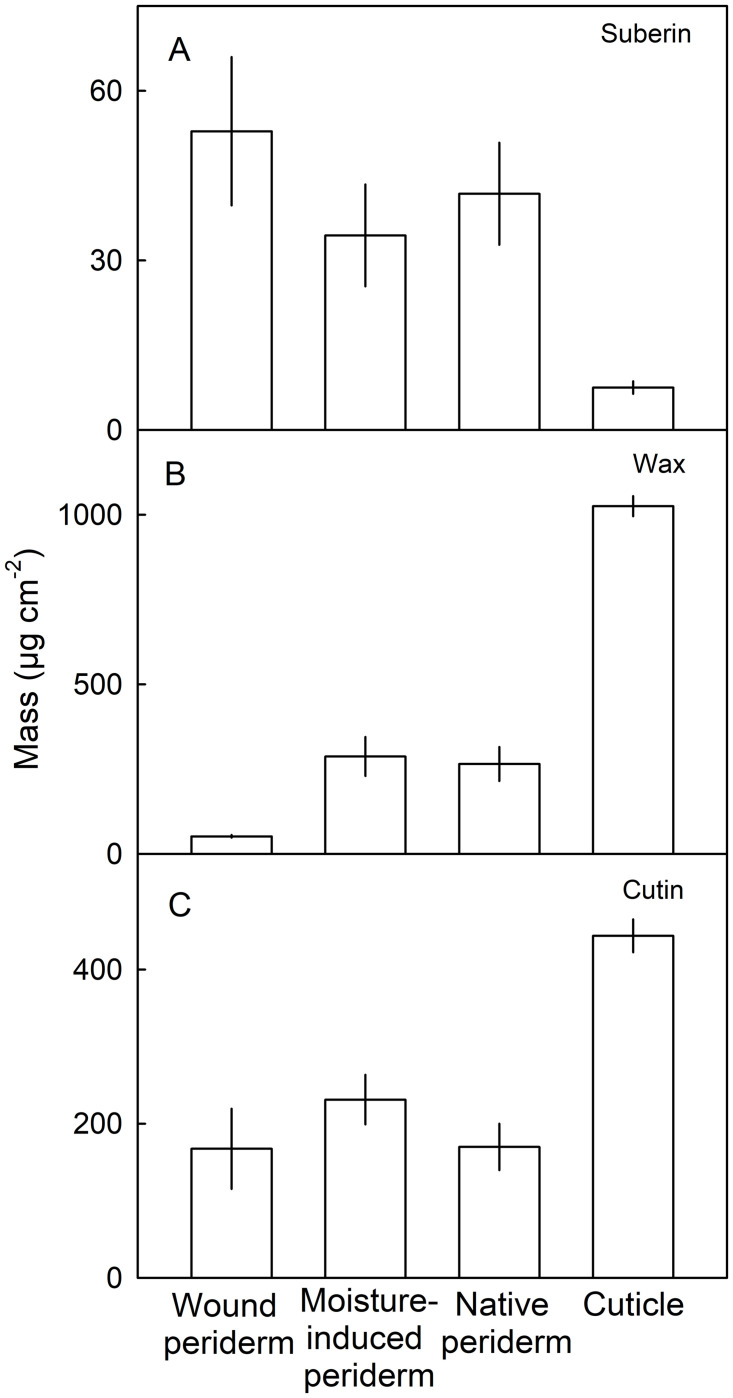
Total masses of suberin (A), wax (B) and cutin (C) in patches of skin of mature apple fruit. Periderm formation in the fruit skin was induced during early fruit development by abrading the cuticle using fine sandpaper (‘Wound periderm’) or by exposing the fruit skin to surface moisture for 12 d between 31 and 43 days after full bloom (DAFB; ‘Moisture-induced periderm’; [22]). The treated patches of skin were excised at maturity 156 DAFB. Periderm from naturally russeted fruit (Native periderm) and cuticles from non-treated, non-russeted fruit (Cuticle) served as controls. Data represent means ± SE of two to three replicates comprising periderms and cuticles of five fruit each.

Total suberin was higher and total wax was lower, in the three periderms compared with in the cuticle. Accordingly, cutin occurred in higher amounts in the cuticle than in any of the three periderms (Fig 8).

**Fig 9:**
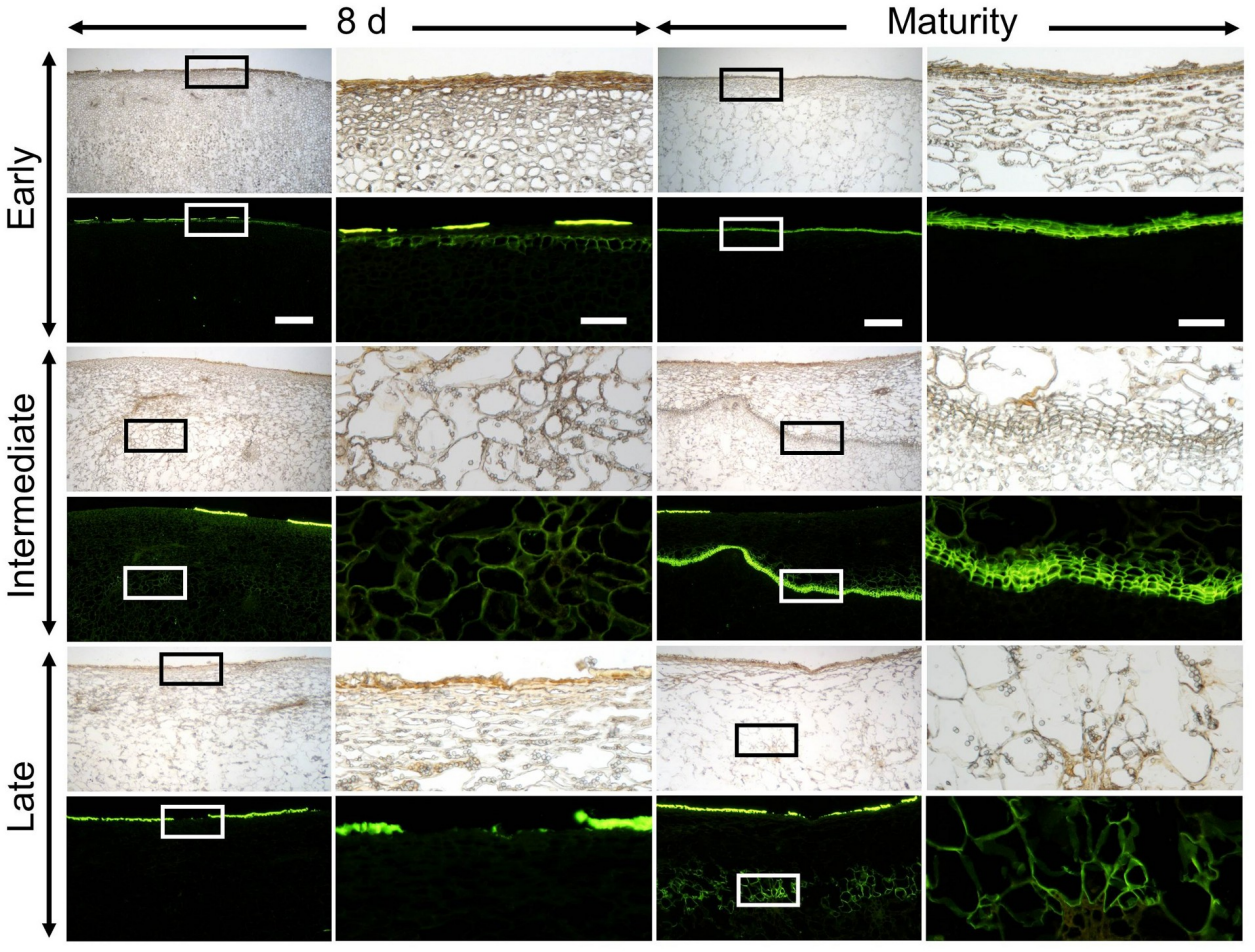
Developmental time course of periderm formation following wounding of ‘Pinova’ apple at 32 days after full bloom (DAFB) (‘early’), 66 DAFB (‘intermediate’) and 93 DAFB (‘late’). For wounding, the cuticle was abraded using fine sandpaper. Cross-sections were prepared 8 d after wounding (left panel) or at maturity (156 DAFB) (right panel). Pairs of micrographs were taken under transmitted white light or incident fluorescent light (filter module U-MWB) after staining with Fluorol Yellow. Fluorol Yellow stains the cuticle and suberized cell walls. Sections were viewed at 20× (scale bar 500 μm, left column) or at 100× (scale bar 100 μm, right column).

Marked differences were found in periderm formation between different stages of fruit development. Wounding during early fruit development (32 DAFB) resulted in a typical periderm characterized by stacked and suberized phellem cells after 8 d of wounding, and which were still visible at maturity (156 DAFB; Fig 9). When wounding occurred at 66 DAFB a layer of cells with suberized cell walls had formed in the cortex within 8 d. At maturity, a typical periderm had developed (Fig 9). Interestingly, following wounding at a late stage of development (93 DAFB) only cells with suberized cell walls had formed in the cortex at maturity, but not a complete periderm (Fig 9).

## Discussion

Our results demonstrate that periderms induced by wounding and by surface moisture are similar and do not differ from periderms found on naturally russeted fruit surface. This conclusion is based on the following arguments.

First, there was no difference in morphology and histology between wound induced and moisture induced periderms and both were similar to those of a native periderm. The skin sections inspected revealed all typical characteristics of a periderm. These include stacks of phellem cells. These cells have suberized cell walls and therefore stain with Fluorol Yellow [30]. The ‘stacked’ arrangement indicates the cells in a stack originate from a single mother cell of the phellogen.

Second, gene expression was similar following wounding and following termination of moisture exposure. Genes related to the synthesis and transport of suberin monomers and transcription factors involved in periderm formation were all upregulated. Those involved in the synthesis and transport of cutin monomers and wax components were downregulated. In an earlier study, Straube et al. [22] observed an upregulated expression of *CYP86B1*, *MYB42*, *ABCG20*, *NAC038*, *NAC058* and *MYB93* in moisture exposed patches of apple skins after termination of the treatment. *CYP86B1* is a key gene involved in the synthesis of very long chain ω-hydroxy and α,ω-dicarboxylic acids, the monomers of suberin [22, 31]. The transcription factor *MYB42* is involved in regulation of lignin synthesis [32]. *ABCG20* is required for the transport of suberin monomers [33]. *NAC038* and *NAC058* are transcription factors of the NAC family that are upregulated in russeted skins of apple [25, 34]. Expression of *MYB93*, another transcription factor, was also expressed in the russeted skin of apples [25, 34]. Its overexpression in *N*. *benthamiana* enhanced the expression of *NAC038* and *NAC058* [34]. Additionally a multispecies gene coexpression analysis highlighted a possible involvement of *NAC038* and *NAC058* in transcriptional regulation of suberin synthesis [35].

Third, there was little difference in composition between the wound induced, moisture induced or native periderms. While cutin and suberin share common monomers, the long chain ω-hydroxy acids (C_20_, C_22_, C_24_) are unique for suberin [22, 36–38]. These dominated in all three periderms. Despite similarity in suberin composition, the wound periderm had a lower wax content compared to native and moisture induced periderms. The reason for this may be the following: the damage caused by abrading the cuticle was so harsh that most of the cuticle was removed and thus the developing periderm on the wounded surface contained no or very much less residual cuticle. In contrast, the native periderms contained significant amounts of dried cuticle residue on the surface [39, 40]. The moisture induced periderm is also expected to contain cuticle residues on the surface as the etiologies of periderm development and periderm morphology are similar to native periderm (Fig 5). The report of Schreiber et al. [41] for potato tubers, that the wound periderm contained 40 to 50% less wax than the native periderm, also supports of our findings.

Fourth, the ontogenies of formation of wound induced periderm and moisture induced periderm were similar. Periderms formed in developing fruit but did not develop in mature fruit. This observation is also consistent with earlier observations [4, 11, 12, 22, 42–44]. Also, Winkler et al. [15] reported that overhead sprinklers induced russet in ‘Elstar’ apples during early fruit development, but not shortly before maturity or at maturity. Apparently, the ability to form a periderm is lost by the later stages of fruit development. A possible explanation to account for this may be a decrease in the rate of growth strain. Towards maturity, the relative area growth rate of the fruit surface decreases continuously. Growth strain represents the main driver of microcracking [9].

The similarity of the periderms induced by wounding or by moisture and native periderms suggests the processes triggering periderm formation are likely similar. In all three periderms, the barrier properties of the cuticle are impaired due to microcracking, the only difference being the reason for the microcracking. While microcracking of the cuticle occurs at the surface, periderm formation begins by a de-differentiation of the subtending hypodermal cells. This requires some sort of signal which connects the two events. Potential signals resulting from impaired barrier properties include: (1) a decreased CO_2_ concentration, (2) an increased O_2_ concentration and (3) a more negative water potential of the flesh due to a more rapid dehydration at the fruit surface [8, 11, 22].

Among those potential signals, the roles of O_2_ and CO_2_ have been studied in kiwifruit and potato tuber. In kiwifruit, wound periderm formation was reduced significantly when O_2_ was eliminated from the storage atmosphere [45]. Similarly, in potato tuber, there was nearly no periderm on the tuber stored at low (0.5 to 1%) O_2_. In contrast, 2 to 4 layers of periderm cell had formed when tubers were stored at ambient (21%) O_2_ concentrations [46]. Based on the observation in kiwifruit, the reduced suberization resulted from decreased activities of phenylalanine ammonia-lyase, peroxidase, catalase, and polyphenol oxidase [45]. Exposure to elevated CO_2_ concentrations (10%) reduced periderm development in potato tuber [47]. To our knowledge, there are no reports of a potential role for a decreased water potential in the tissue surrounding a microcracked cuticle, in triggering periderm formation.

## Conclusion

Periderms induced by wounding or moisture are similar from morphological, histological, compositional and molecular perspectives. Thus, the signal(s) linking the impaired barrier properties to the differentiation of a periderm in the hypodermis is likely to be the same after wounding and after moisture induced microcracking. These findings have important implications for experimental research. The data presented herein justify the use of wounding to study the relationship between the impaired barrier properties of the cuticle due to formation of microcracks and the beginning of periderm formation in the hypodermis, some cell layers below. The search for the linking signal may now begin.

## Supporting information

S1 Table Selected transcription factors and genes analyzed in the present study. (DOCX)

S2 Table Primer sequences of the genes analyzed in the present study. (DOCX)

S1 Dataset Excel file containing all data produced in figures throughout the manuscript. (XLSX)
