# Supplementary material for: Apple fruit periderms (russeting) induced by wounding or by moisture have the same histologies, chemistries and gene expressions
Source: PLoS One. 2022 Sep 29;17(9):e0274733. doi: 10.1371/journal.pone.0274733 (PMC9522254; doi:10.1371/journal.pone.0274733)
Supplement: S1 Table — (DOCX) [file pone.0274733.s001.docx]

| **S1 Table.** Selected transcription factors and genes analyzed in the present study. | | | | |
| --- | --- | --- | --- | --- |
| Gene name | Accession | AGI locus code | Description | Reference |
| Suberin and lignin related | | | | |
| *MYB93* | MDP0000320772 | AT1G34670.1 | MYB domain protein 93, positive regulator of suberin biosynthesis | [48] |
| *MYB42* | MDP0000787808 | AT4G12350.1 | MYB domain protein 42, involved in secondary cell wall biosynthesis and regulation of lignin synthesis | [49,50] |
| *CYP86B1* | MDP0000306273 | AT5G23190.1 | Cytochrome P450, family 86, subfamily B, polypeptide 1, involved in the synthesis of very long chain *ω*-hydroxyacid and *α*,*ω*-dicarboxylic acid in suberin polyester | [51] |
| *ABCG20* | MDP0000265619 | AT3G53510 | ATP-binding cassette G20, involved in transport of aliphatic suberin polymer precursors | [52] |
| *NAC038* | MDP0000232008 | AT2G24430.1 | NAC domain containing protein 38 | uncharacterized |
| *NAC058* | MDP0000130785 | AT3G18400.1 | NAC domain containing protein 58 | uncharacterized |
| Cutin and wax related | | | | |
| *SHN3* | MDP0000178263 | AT5G25390 | Positive transcriptional regulator of cuticle synthesis | [53] |
| *GPAT6* | MDP0000479163 | AT2G38110.1 | Glycerol-3-phosphate acyl transferase 6, involved in synthesis of cutin monomers | [54] |
| *FDH, KCS10* | MDP0000235280 | AT2G26250.1 | FIDDLEHEAD, 3-Ketoacly-CoA synthase 10, involved in the long-chain lipid synthesis | [55] |
| *CER6* | MDP0000392495 | AT1G68530.1 | 3-Ketoacly-CoA-synthase 6, involved in the biosynthesis of very long chain fatty acids (VLCFAs) | [56] |
| *WSD1* | MDP0000701887 | AT5G37300.1 | Wax ester synthase/Acyl-coenzyme A: Diacylglycerol acyltransferase, involved in the synthesis of wax esters and diacylglycerol acyltransfer activity | [57] |
| *ABCG11* | MDP0000200335 | AT1G17840.1 | ABCG11, white-brown complex homolog protein 11, transport of cuticular lipids to the extracellular matrix | [58] |
| Table was adopted from Straube et al. [22] | | | | |

**References**

22. Straube J, Chen YH, Khanal BP, Shumbusho A, Zeisler-Diehl V, Suresh K, et al. Russeting in apple is initiated after exposure to

moisture ends: Molecular and biochemical evidence. Plants. 2021; 10:65. https://doi.org/10.3390/plants10010065

1. Legay S, Guerriero G, André C, Guignard C, Cocco E, Charton S, et al. *MdMyb93* is a regulator of suberin deposition in russeted apple fruit skins. New Phytol. 2016; 212:977-991. https://doi.org/10.1111/nph.14170
2. Zhong R, Lee C, Zhou J, McCarthy RL, Ye ZH. A battery of transcription factors involved in the regulation of secondary cell wall biosynthesis in Arabidopsis. Plant Cell. 2008; 20:2763-2782. https://doi.org/10.1105/tpc.108.061325
3. Geng P, Zhang S, Liu J, Zhao C, Wu J, Cao Y, et al. *MYB20*, *MYB42*, *MYB43*, and *MYB85* regulate phenylalanine and lignin biosynthesis during secondary cell wall formation. Plant Physiol. 2020; 182:1272-1283. https://doi.org/10.1104/pp.19.01070
4. Compagnon V, Diehl P, Benveniste I, Meyer D, Schaller H, Schreiber L, et al. *CYP86B1* is required for very long chain omega-hydroxyacid and alpha, omega -dicarboxylic acid synthesis in root and seed suberin polyester. Plant Physiol. 2009; 150:1831-1843. https://doi.org/10.1104/pp.109.141408
5. Yadav V, Molina I, Ranathunge K, Castillo IQ, Rothstein SJ, Reed JW. *ABCG* transporters are required for suberin and pollen wall extracellular barriers in Arabidopsis. Plant Cell. 2014; 26:3569-3588. https://doi.org/10.1105/tpc.114.129049
6. Aharoni A, Dixit S, Jetter R, Thoenes E, van Arkel G, Pereira A. The *SHINE* clade of AP2 domain transcription factors activates wax biosynthesis, alters cuticle properties, and confers drought tolerance when overexpressed in *Arabidopsis*. Plant Cell. 2004; 16:2463-2480. https://doi.org/10.1105/tpc.104.022897
7. Petit J, Bres C, Mauxion JP, Tai FWJ, Martin LBB, Fich EA, et al. The Glycerol-3-Phosphate Acyltransferase *GPAT6* from tomato plays a central role in fruit cutin biosynthesis. Plant Physiol. 2016; 171:894-913. https://doi.org/10.1104/pp.16.00409
8. Pruitt RE, Vielle-Calzada JP, Ploense SE, Grossniklaus U, Lolle SJ. *FIDDLEHEAD*, a gene required to suppress epidermal cell interactions in Arabidopsis, encodes a putative lipid biosynthetic enzyme. Proc Natl Acad Sci USA. 2000; 97:1311-1316. https://doi.org/10.1073/pnas.97.3.1311
9. Millar AA, Clemens S, Zachgo S, Giblin EM, Taylor DC, Kunst L. *CUT1*, an *Arabidopsis* gene required for cuticular wax biosynthesis and pollen fertility, encodes a very-long-chain fatty acid condensing enzyme. Plant Cell. 1999; 11:825-838. https://doi.org/10.1105/tpc.11.5.825
10. Li F, Wu X, Lam P, Bird D, Zheng H, Samuels L, et al. Identification of the wax ester synthase/acyl-coenzyme A: Diacylglycerol acyltransferase *WSD1* required for stem wax ester biosynthesis in *Arabidopsis*. Plant Physiol. 2008; 148:97-107. https://doi.org/10.1104/pp.108.123471
11. Bird D, Beisson F, Brigham A, Shin J, Greer S, Jetter R, et al. Characterization of *Arabidopsis* *ABCG11/WBC11*, an ATP binding cassette (ABC) transporter that is required for cuticular lipid secretion. Plant J. 2007; 52:485-498. https://doi.org/10.1111/j.1365-313X.2007.03252.x
