# Supplementary material for: Apple fruit periderms (russeting) induced by wounding or by moisture have the same histologies, chemistries and gene expressions
Source: PLoS One. 2022 Sep 29;17(9):e0274733. doi: 10.1371/journal.pone.0274733 (PMC9522254; doi:10.1371/journal.pone.0274733)
Supplement: S2 Table — (DOCX) [file pone.0274733.s002.docx]

| **S2 Table.** Primers sequences of the genes analyzed in the present study | | | | | |
| --- | --- | --- | --- | --- | --- |
| Gene name | Accession | Primer sequence (5' to 3') | | PCR efficiency (%) | Reference |
|  |  | Forward Primer | Reverse Primer |  |  |
| *ABCG11* | MDP0000200335 | TGGCGGGTTTCCTTCTTTCA | CACAAATGCAGTAACGCCGT | 98.7 | [22] |
| *ABCG20* | MDP0000265619 | ACTGGGCATGGACAACAACA | ATTTTCCCGACCCACTTGCT | 102.9 | [22] |
| *CER6* | MDP0000392495 | AGCAACAACCCTAAGAGCGT | GTTGGGCGGAATGTAGTGGA | 85.0 | [22] |
| *CYP86B1* | MDP0000306273 | CGCTTTGTGACCCCATCC | AATGACGTCTTCCGCAAACT | 109.3 | [25] |
| *eF-1alpha* | AJ223969.1 | ACTGTTCCTGTTGGACGTGTTG | TGGAGTTGGAAGCAACGTACCC | 93.0 | [25] |
| *GPAT6* | MDP0000479163 | TCTTGAACCAGCTACCGTCG | AATCCCAAAGTCCCAGCCAA | 91.0 | [22] |
| *KCS10* | MDP0000235280 | TGCTGAGGTGGGAAGTTTGA | ACACCAAAGAACCCTAGCACA | 91.6 | [22] |
| *MYB42* | MDP0000787808 | CCTTGGCAATAGGTGGTCGA | TGATGTGCGTGTTCCAGTGA | 94.2 | [22] |
| *MYB93* | MDP0000320772 | TGGACAAACTATCTTAGGCCGG | GTTGCCGAGGATGGAATGGA | 102.5 | [22] |
| *NAC038* | MDP0000232008 | CGGCGGATCATCAAGTAGCA | AAACCCTCCTCCTCCTCCAA | 84.6 | [22] |
| *NAC058* | MDP0000130785 | AGCCACAACAAGCAACAACA | TTTGGCAGCTCGATGTCTCC | 90.7 | [22] |
| *PDI* | MDP0000233444 | TGCTGTACACAGCCAACGAT | CATCTTTAGCGGCGTTATCC | 100.6 | [22,59] |
| *SHN3* | MDP0000178263 | GGGACGTTTGAGACAGCAGA | TTTTGGTCGGTGGCAGGTTT | 93.6 | [22] |
| *WSD1* | MDP0000701887 | AGAAATGGTCAAACCCGACA | AGACGAAGTCAAGCGCATTT | 91.1 | [25] |
| Table was adopted from Straube et al. [22] | | | | | |

References

22. Straube J, Chen YH, Khanal BP, Shumbusho A, Zeisler-Diehl V, Suresh K, et al. Russeting in apple is initiated after exposure to

moisture ends: Molecular and biochemical evidence. Plants. 2021; 10:65. https://doi.org/10.3390/plants10010065

25. Legay S, Guerriero G, Deleruelle A, Lateur M, Evers D, André CM, et al. Apple russeting as seen through the RNA-seq lens: Strong alterations

in the exocarp cell wall. Plant Mol Biol. 2015; 88:21-40. https://doi.org/10.1007/s11103-015-0303-4

59. Storch TT, Pegoraro C, Finatto T, Quecini V, Rombaldi CV, Girardi CL. Identification of a novel reference gene for apple transcriptional

profiling under postharvest conditions, PLoS ONE. 2015; 10:e0120599. https://doi.org/10.1371/journal.pone.0120599
